# Effects of captions, transcripts and reminders on learning and perceptions of lecture capture

**DOI:** 10.1186/s41239-022-00327-9

**Published:** 2022-04-26

**Authors:** Eleanor J. Dommett, Larisa M. Dinu, Wijnand Van Tilburg, Samuel Keightley, Benjamin Gardner

**Affiliations:** 1 Department of Psychology, Institute of Psychiatry, Psychology and Neuroscience, London, SE5 8AF UK; 2 Department of Psychology, University of Essex, Colchester Campus, Colchester, CO4 3SQ UK; 3 Centre for Technology Enhanced Learning, King’s College London, London, SE1 9NH UK

**Keywords:** Analytics, Lecture capture, Student performance

## Abstract

Lecture capture is popular within Higher Education, but previous research suggests that students do not always optimally select content to review, nor do they make the most of specific functions. In the current study conducted in the 2019/20 academic year, we used a repeated-measures crossover design to establish the effects of transcripts with closed captioning, and email reminders, on use (self-reported and system analytics), perceptions of lecture capture and student performance, as measured by multiple-choice question (MCQ) tests designed to assess the module learning outcomes. System analytics (N = 129) and survey data (N = 42) were collected from students alongside qualitative data from semi-structured interviews (N = 8). We found that students value lecture capture highly, but do not access it extensively during the teaching period. The availability of transcripts and closed captions did not impact the amount of capture use or performance on MCQ tests, but did result in more positive perceptions of capture, including increased likelihood of recommending it to others. The use of email reminders referring students to specific segments of capture and reminding them of the functionality had no impact on any measure, although qualitative data suggested that the content of reminders may be used in revision rather than during the teaching period, which fell outside the period we investigated. Collectively, these data suggest that the use of captions and transcripts may be beneficial to students by allowing dual processing of visual and audio content, and a searchable resource to help consolidate their learning but there is little evidence to support reminders.

**Supplementary Information:**

The online version contains supplementary material available at 10.1186/s41239-022-00327-9.

## Introduction

Lectures are engrained within Higher Education (HE) where they provide an efficient way to teach large numbers of students simultaneously (Behr, 1988; Dommett, Gardner, et al., 2019; Dommett et al., 2020; Dommett, van Tilburg, et al., 2019). Students value lectures highly (Dommett et al., 2020). This became even more apparent during the COVID-19 pandemic, when in-person lectures were unavailable for many students. When surveyed on what students had missed most during the pandemic, in-person lectures were ranked second, only falling behind missing out on friends and social activities and critically, were missed much more than other types of in-person teaching (Ezarik, 2021). This is perhaps unsurprising when lectures are perceived by students as providing subject overviews and delivering core knowledge and understanding (Dommett, Gardner, et al., 2019; Dommett, van Tilburg, et al., 2019) which can then be consolidated in more active learning sessions. In terms of skills development, lectures have been associated with independent thinking, problem solving (Covill, 2011), modelling expert behaviour (Feldon, 2010), and building links between material (Kirkpatrick, 1990).

The recording of live lectures, referred to as lecture capture (LC), is common practice, with recordings (e.g., audio and slides, audio-only, video and slides) typically made available via virtual learning environments (VLEs) (Deal, 2007; Traphagan, 2005; Woo et al., 2008) after the live event. The rationale behind capture is rarely stated, but it is thought to increase inclusivity; for example, making learning more manageable for those for whom the instructional language is not their first language or who experience specific learning or sensory differences (Kushnir et al., 2011; Leadbeater et al., 2013; Taplin et al., 2014). Students with disabilities do value the capture more (Dommett et al., 2020) but even in the absence of disabilities, students feel the availability of capture supports their wellbeing (Dommett, Gardner, et al., 2019; Dommett, van Tilburg, et al., 2019). The COVID-19 pandemic has also shown how lecture capture can support remote learners who cannot attend a live lecture, further cementing a position for lecture capture technologies within Higher Education.

Despite students valuing lectures and capture highly, there is variety in how lecture capture is used by students. Even before the COVID-19 pandemic, many students used capture to substitute for in-person attendance, whereas others watch the entire lecture after attending in person, and others dip in and out to review complex points (Dommett et al., 2020). Substitution of attendance is a contentious issue and is linked to both changes in student performance (Edwards & Clinton, 2018) and staff concerns (Dommett, Gardner, et al., 2019; Dommett, van Tilburg, et al., 2019). However, repeated watching of the entire lecture to make notes, as has been observed (Dommett et al., 2020), can add significantly to the student workload. Given that higher workload is associated with stress and negative wellbeing in students (Smith, 2019), it is important to ensure students use their time optimally. It is therefore likely that selectively targeting specific areas of the captured lectures to playback and review points missed or complex material may be preferable (Gorissen et al., 2012; Gosper et al., 2010; Groen et al., 2016; Watt et al., 2014). This can be challenging for students, especially those early in their studies, who may still be adapting to higher education practices. Our own experience is that students do struggle to pick out which sections are critical and to identify complexities, meaning it may be beneficial to identify these sections for students initially.

Students typically have limited knowledge of the full functionality of capture tools when using captures of live lectures after the lecture has occurred. For example, a study looking at use of Echo360 capture software found that most were aware of how to adjust playback speed and pause the video, but were unaware of how to bookmark and make study notes within the software, despite feeling these features would be useful (Dommett et al., 2020). Indeed, in this study, students were provided with documentation on how to use capture through the VLE, at the same point where they accessed captures, but still had limited knowledge, suggesting it is insufficient to provide guidance resources online.

Specific functionality could add to the utility of lecture capture for students, but only if used successfully. One such function is the addition of closed captions and associated transcripts to the capture. This is a particularly important feature given recent legislation about accessibility of websites, including VLEs in the UK and Europe (JISC, 2018). Captioning and transcripts are thought to support students at risk of struggling due to hearing impairment, second language or conditions such as Autism Spectrum Disorder (Alsalamah, 2020; Kent et al., 2018). However, a case can also be made for captioning and transcripts to be beneficial to a broader cohort, drawing on the Cognitive Theory of Multimedia Learning (Mayer, 2014). This theory assumes that people have separate channels for processing visual and verbal information, but have a limited capacity in working memory for each of these channels, and so must actively process information for meaningful learning to occur. The theory proposes that captioned lectures (whether live or pre-recorded) can provide a dual channel approach to processing, with the spoken word (verbal) and caption (visual) operating together. Yet, research has largely focused on use of closed captioning and transcripts for supporting second language development (Balci et al., 2020) rather than its broader benefits. Some research has demonstrated that captioning and transcripts can be helpful to students more broadly, especially where the lecturer has a heavy accent or audio quality is poor (Tisdell & Loch, 2017), but research in this area is lacking. The few studies attempting to evaluate the utility of captioning and transcripts on student performance have given mixed results, variously showing benefits (Ranchal et al., 2013; Taylor, 2009), no effects (Allen & Katz, 2011) and adverse effects (Ritzhaupt et al., 2015).

Recent legislation around accessibility of VLEs and shifts in teaching delivery during the COVID-19 pandemic, should catalyse further research into functionality to support inclusivity, including captions and transcript. Many universities are expected to continue with online modes of teaching post-pandemic, with the most common teaching mode being blended delivery (Maguire et al., 2020), which will likely include pre-recorded videoed content, including lectures. Changes induced by the COVID-19 pandemic have thus accelerated pre-existing shifts towards blended, online learning (Murphy, 2020).

In the current study, we aimed to investigate two specific elements of lecture capture practice to add to the burgeoning body of research in this field. Firstly, because previous research indicates that students value capture highly but are often unaware of the functional capabilities and may not use it efficiently, we aimed to investigate ways of improving their use of lecture capture. Specifically, we aimed to investigate the impact of push reminders on students’ use of and opinions about lecture capture, as well as measuring their performance on tests of module content. Secondly, we aimed to investigate the impact of captioning and transcripts on students’ lecture capture use, opinions and performance. For the capture software we used in this study (Echo360), transcripts provide the closed captioning, so it was not possible to have captions without transcripts. However, once transcripts are available, it is possible to toggle captions on and off. For this reason, we consider captions and transcripts together.

## Materials and methods

### Case study context

This research was conducted at a large UK London Russell Group university, which has been using lecture capture (via the Echo360 platform) since September 2015. Lecture capture use at the institution is part of an opt-out policy whereby all lectures are recorded unless staff make a case, in advance, to senior faculty management to opt out. Lectures are typically captured using video, audio and slide capture in larger spaces (e.g., lecture theatres with capacity > 100) and audio and slide capture in small spaces. At the time of the study (2019), captions and transcripts were not automatically made available with lecture capture, although the functionality was available within Echo360. Given that captions and transcripts often require manual corrections, which can involve a considerable cost, both time and financial, it was deemed necessary to examine whether this additional functionality was a benefit to students, prior to enabling it across the institution. It is noteworthy that at this institution Echo360 LC software is typically only used for capturing live lectures, where students would be expected to attend in person. Alternative software is used to pre-record videos for use in teaching, in the absence of a live audience.

This specific study was conducted in the context of a first-year undergraduate Social Psychology module. The module is only open to students studying for a BSc Psychology and is a core module in the university’s British Psychology Society accredited qualification. A copy of the module learning outcomes can be found in Additional file 1. The module adopts the same overall structure as other modules within the programme. It runs during the first semester of the academic year (Sept–Dec) and therefore, in the academic year under study (2019/20), took place prior to the COVID-induced transition to online learning (March 2020). Phase 1 was conducted during and immediately after the teaching period (Oct–Dec 2019) and Phase 2 conducted in the second semester (Jan–Mar 2020, before the university was closed due to COVID-19 restrictions). Participants were drawn from a module cohort of 271 students. The module was structured to include two hours of lectures each week along with either a seminar session or a practical class over an eleven-calendar-week period. Each week covered one of ten specific topics, with a Reading Week in the middle (late Oct-early Nov 2019) during which there was no teaching. The module was assessed by a coursework essay, submitted immediately after teaching finished (Dec 2019), and a multiple-choice question examination (Jan 2020), both of which are designed to assess the learning outcomes collectively. The structure of the module made a crossover design possible, as detailed below.

### Overview of methodology

We opted to use a mixed methods research methodology, which typically yields greater insights than from qualitative or quantitative methods alone (Creswell & Creswell, 2017). We opted to use a sequential explanatory design, which is commonly used in education research (Creswell et al., 2003) and has previously been successful when considering lecture capture (Dommett et al., Dommett, Gardner, et al., 2019). Quantitative data was collected in Phase 1 in the form of data from both the lecture capture system and students via a survey, about their use and perception of Lecture Capture as well as a measure of performance. Qualitative data was collected in Phase 2 using semi-structured interviews with students, to help explain or elaborate on the quantitative findings. This approach ensured that the quantitative data provided a general response to the research questions with the qualitative data assisting in explaining the quantitative results (Creswell, 2014).

### Phase 1: quantitative phase

#### Participants and recruitment

All students registered on the module were invited to take part during their induction week (mid-late Sept). During this week, students underwent a digital education induction which included activities related to various platforms that they would subsequently use as part of their studies. This included the VLE (a Moodle-based platform) and a surveying tool (Qualtrics). During the digital education session, students were provided with information about the study and an online consent form hosted in Qualtrics. Those wishing to give informed consent completed this form and provided identifiable information (Name, Email Address, Student ID) to allow them to be allocated to study groups on the VLE. Students who completed both available online surveys were offered a prize draw entry to win one of three (£100, £50, £25) shopping vouchers. Of the 271 students registered on the module, 133 (49%) agreed to participate. Ethical approval was obtained from the Institutional Ethical Review Committee (LRS-18/19–13,686).

#### Design

A crossover factorial design was used to assess the impact of two independent variables (IVs): (1) availability of captions and transcripts and (2) use of emailed reminders. This design reduces the risk of confounding variables impacting results because all interventions are tested in the same participants. This also means that fewer overall participants are required (Heeson, 2020). This approach is advocated in other disciplines, such as medicine, and recent research shows that similar trial designs can be effective in education research (Churches et al., 2020). This was also deemed the most ethical design by our institution because all students participating had access to experimental conditions (i.e., transcripts, captions and email reminders) that would be expected to improve their learning experience and performance.

**Fig. 1:**
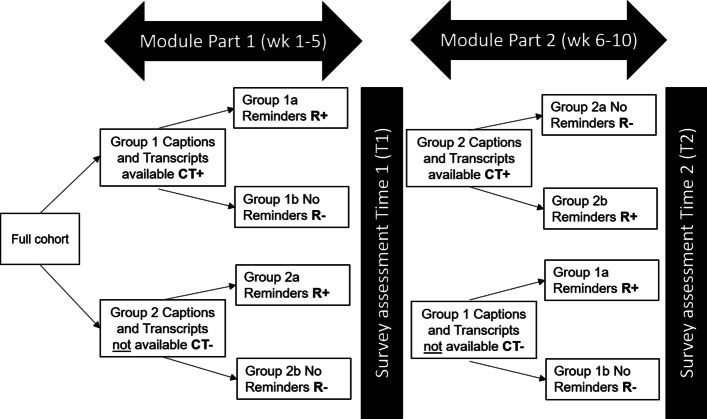
An overview of the quantitative phase of data collection indicating alignment to the module teaching and the two survey points

Students were randomly allocated, using a random number generator in Excel, to one of two groups for the caption and transcript variable such that Group 1 had access to these (CT +), and Group 2 did not (CT−), during the first half of the semester, covering five of the ten module topics. This function was enabled through group-specific links on the VLE. Groups were reversed at Reading Week, such that in the second half of the semester, Group 2 had access to captions and transcripts for the remaining five topics and Group 1 did not. Restricted randomisation was used to ensure group sizes were as similar as possible. Within each CT group, there was further restricted random allocation into two reminder groups, with one receiving reminders (R +) and one not (R−). This grouping was also reversed at Reading Week. All participants were therefore exposed at some point to captions and transcripts with and without reminders. After data had been collected, all students were given access to lecture capture with captions and transcripts if they wished to use it in preparation for their exam. This design is summarised in Fig. 1.

Live lectures took place on Wednesdays and lecture capture for all students was made available from afternoon on the following day. The delay was necessary to ensure that the transcripts and captioning were accurate. Reminders, sent to R + students only, consisted of a weekly email sent on Fridays reminding students to review the weekly lecture capture using the various functions available, with links to instructions on how to use specific functions, and, for CT + students only, reference to transcripts and captions. These emails also directed students to up to three sections, using time codes, recommended by the module leader as particularly important or challenging. Those in the R − group did not receive these messages. Given previous research suggesting that email reminders increase study time at weekends only (O’Connell & Lang, 2018), and that our students attended subsequent teaching activities (seminars or practicals) the following Monday or Tuesday, we deemed Fridays the ideal day to send reminders.

#### Measures

Dependent variables were drawn from both the lecture capture system analytics and online surveys completed by students. The dependent variables were measured at two time points: (1) during Reading Week, before reversal of exposure (Time 1); and during the week after teaching of the final module topic had finished (Time 2). Lecture capture system analytics included the percentage of the capture viewed for each week and the number of video views. The number of video views shows the number of times a student accessed the capture for a specific topic. For example, three video views would indicate that the capture for a specific lecture was accessed three times. These data were missing for four students who withdrew or interrupted their degree studies during the semester, giving a final analytics sample size of N = 129. The online survey included ten multiple choice questions (MCQs) similar to those in the real module exam format and therefore designed to assess improvement against the module learning outcomes for specific topics, an example of which is given in Additional file 2. There were two questions for each topic of study in the five weeks prior to the survey. These were used to assess student learning, as a measure of performance. Students were advised that these were similar in style to the exam. In addition, students were asked to indicate which of the five possible topics they had used the capture for, and which lecture capture functions they had used. They were also asked to rate their agreement with statements relating to ease of use of lecture capture, capture supporting learning and recommending capture to friends (e.g., “I have found lecture capture easy to use.” 1 = strongly disagree, 5 = strongly agree). Finally, they were asked to indicate usefulness of captions and transcripts and reminders where they had access to them (1 = Not at all useful, 5 = Extremely useful). Of 129 participants who did not withdraw from their degree programme, 42 (33%) completed both surveys.

#### Data analysis

Lecture capture analytics data (video views and percentage viewed) was extracted from the capture platform and transferred into SPSS for analysis. The average number of video views and percentage viewed was calculated for each half of the semester for each participant. Paired sample *t*-tests were used to compare measures between the first and second half of the semester. Within each period, independent sample *t*-tests were used to compare the impact of captions and transcripts and reminders for all students.

Survey data were extracted from Qualtrics, directly into SPSS for analysis. In addition to descriptive characteristics, independent sample *t*-tests were used to examine the impact of captions and transcripts and reminders across the whole semester for continuous variables. For categorical measures of the functions used within the lecture capture platform, the within subjects Chi-Square test (McNemar test) was used.

### Phase 2: qualitative phase

#### Participants and Recruitment

During the second semester, after completion of the module, Phase 2 began. Participants in the quantitative phase were invited via email, and adverts on the VLE, to take part in in-person or online semi-structured interviews about their experiences of using lecture capture on this specific module. We had originally intended to conduct all interviews in person and sample 12–15 students, based on research indicating that the vast majority of analytic insights from qualitative interviews emerge from the first 10–12 sampled participants (Guest et al., 2006; Morgan et al., 2002). However, due to the COVID-19 pandemic, interviews were switched to online via MS Teams and the final sample size was reduced due to limited student availability. During this period, students were completing their final teaching weeks online but availability to captured lectures remained the same as before the university closure. In total, 8 interviews were conducted. Participants were given a £10 voucher on interview completion. The quantitative and qualitative data were not linked. This meant that we could not identify specific details of LC use in the interviewed students. This was a deliberate decision because the quantitative data included learning outcome (performance) data which, if identifiable to the interviewer, may have impacted on students’ willingness to be interviewed.

#### Design

Semi-structured interviews were deemed suitable because they provide greater flexibility than structured interviews for probing areas of apparent pertinence to participants (Drever, 1995). The interview schedule was designed to capture students’ perceptions of lectures, use of lecture capture, and the impact of captions, transcripts and reminders. An initial interview schedule drafted prior to the start of the study was refined following analysis of quantitative data, in-keeping with the sequential explanatory design. The full schedule is shown in Additional file 2.

#### Data analysis

Verbatim automated transcripts, generated using Otter.ai, were checked for mistakes and corrected manually. Inductive Thematic Analysis was conducted by two authors and followed a six-stage process, involving: data familiarisation; coding; theme extraction; theme review; theme naming; and narrative analysis (Braun & Clarke, 2006). Both authors independently read and reread transcribed interviews for four participants to familiarise themselves with the data and to note any initial points of analysis. A preliminary, inductively-derived thematic framework was co-constructed through discussion among these coders to enable a detailed coding process, and theme extraction. The discussion process allowed for the incorporation of analytical insights independently reached by multiple coders and insights uniquely contributed by one coder, and for the resolution of any disagreements between coders. Coding and theme extraction involved assigning conceptual labels to ‘events’ within the data, with multiple labels assigned to a single comment where appropriate. Coded data items were collated with relevant extracts so that a review of themes could be conducted. Each coder then independently coded the remaining four interviews and all analyses were collated and themes reviewed. This involved identifying links between conceptual labels and higher-order themes, and constantly refining the labels, themes and framework to best reflect and organise emergent insights. Naming themes involved development of a detailed definition and analysis of theme content, and identification of concise theme labels. Both coders agreed upon the final coding framework and data extracts and verified that themes were valid and coherent representations of the data.

### Researcher positionality

The first author on this paper (EJD) has been actively researching education technology for over a decade. This author, along with the two other academic staff authors (WvT and BG), has been conducting research focusing on lecture capture and video tools for over five years, investigating staff and student attitudes to lectures and their capture both from a teaching and learning perspective and a policy point of view. Both of these academic authors (BG and WvT) taught on the module with (BG) also the module leader. Given their involvement in the module, neither of these authors was involved in data collection or coding. The remaining two authors were a postgraduate student, with no prior history of investigating education methods, and a graduate research assistant, who had previously worked on a large scale study examining online learning. These authors had no prior contact with the students on the module and conducted interviews and coding. All academic staff involved in the study are current users of Lecture Capture to capture their live lectures and the remaining authors had used the tool as students. All authors are psychologists by training. In conducting this research, the authors wanted to better understand how to encourage effective and efficient use of capture in students by establishing what methods may work, and which may not, in order to ensure efforts made to support teaching and learning are in the right areas.

## Results

### Phase 1: quantitative outcomes

#### System analytics: the impact of transcripts and reminders on lecture capture use

All students studying the module had access to capture. Across all those participating in the study who remained on their degree programme (N = 129), the mean number of video views per week during the first half of the semester (Time 1) was 0.73 (*SD* = 0.87) indicating that students were not accessing the capture for all weeks. This remained low in the second part of the semester (Time 2; *M* = 0.52, *SD* = 1.06). The average percentage of the video watched was 23.7% (*SD* = 28.51%) at Time 1 and 18.1% (*SD* = 32.16%) at Time 2, showing that students did not watch videos in full when they accessed them. Between Time 1 and Time 2, there was a significant decrease in the number of views (*t *(128) = 2.299, *p* = 0.023, 95% CI [0.03, 0.40], *d* = 0.202) and percentage of video viewed (*t *(128) = 2.064, *p* = 0.041, 95% CI [0.23%, 11.00%], *d* = 0.182). Given the overall differences between the two time points, we opted to analyse these two periods separately. Table 1 shows that at Time 1, there was no impact of CT + /CT − grouping on either measure. However, at Time 2, CT + students had increased video views and percentage viewed when compared to CT −. Table 2 reveals no impact of reminders during either period.

**Table 1:** The impact on transcript availability on video views and percentage viewed

	CT − Mean (SD)	CT + Mean (SD)	Test statistic *t* (df)	Significance	Effect size (*d*)
Time 1					
Number of views	0.60 (0.82)	0.87 (0.89)	1.799 (127)	0.074	0.317
Percentage viewed	20.49 (25.62)	27.01 (31.02)	1.301 (127)	0.195	0.229
Time 2					
Number of views	0.10 (.38)	0.92 (1.33)	4.790 (74.43)	< 0.001	0.838
Percentage viewed	8.00 (22.71)	28.06 (36.85)	3.727 (106.77)	< 0.001	0.654

**Table 2:** The impact on reminders on video views and percentage viewed

	R − Mean (SD)	R + Mean (SD)	Test statistic *t* (df)	Significance	Effect size (*d*)
Time 1					
Number of views	0.79 (0.95)	0.67 (0.77)	0.848 (127)	0.398	0.149
Percentage viewed	26.93 (29.72)	20.26 (26.96)	1.337 (127)	0.185	0.235
Time 2					
Number of views	0.51 (1.11)	0.52 (1.02)	0.085(127)	0.993	0.015
Percentage Viewed	14.65 (20.01)	21.31 (34.73)	1.186 (125.72)	0.238	0.208

Note that this pattern of significance is maintained when only the subset of participants who completed the survey are analysed (N = 42)

#### The impact of transcripts and reminders on use and student views of capture

Data from the 42 students who completed both surveys were used to determine specific use traits and views of the lecture capture. The number of topics (maximum five) for which they had viewed capture did not differ between the CT + (*M* = 2.40, *SD* = 1.69) and CT − conditions (*M* = 2.14, *SD* = 1.63), *t* (41) = 1.026, *p* = 0.311, 95% CI [− 0.25, 0.78], *d* = 0.158. CT condition also had no impact on the ease of using lecture capture (CT + *M* = 4.77, *SD* = 0.43, CT − *M* = 4.65, *SD* = 0.66, *t* (30) = 1.278, *p* = 0.211, 95% CI [− 0.08, 0.34], *d* = 0.229. There was a trend towards CT + (*M* = 4.90, *SD* = 0.30) compared to CT- (*M* = 4.71, *SD* = 0.46) impacting on how much students perceived capture to support learning, *t* (30) = 1.985, *p* = 0.056, 95% CI [− 0.01, 39], *d* = 0.356, with availability of captions and transcripts resulting in slightly higher learning support ratings. Those in the CT + group were more likely to state that they would recommend LC to a friend (CT + *M* = 4.93, *SD* = 0.25; CT − *M* = 4.74, *SD* = 0.51; *t* (30) = 2.257, *p* = 0.031, 95% CI [0.02, 0.37], *d* = 0.405).

Table 3 shows the functions used by students according to their CT grouping. Most students used the time bar to visit specific sections, pausing and restarting, and adjusting speed, but fewer utilised bookmarking or study notes. McNemar Chi-Square tests comparing functionality found no differences between the two groups.

**Table 3:** Percentage of students using specific functions of lecture capture (N = 38)

Function	CT- (% using)	CT + (% using)	Test statistics, *χ*^*2*^* (df)*	Significance
Time bar	68.4	60.5	0.813 (1)	*0.607*
Pause/Restart	71.1	81.6	3.317 (1)	*0.344*
Speed	57.9	63.2	7.819 (1)	*0.754*
Bookmarking	10.5	5.3	3.493 (1)	*0.625*
Study notes	39.5	39.5	17.036 (1)	*1.00*

**Fig. 2:**
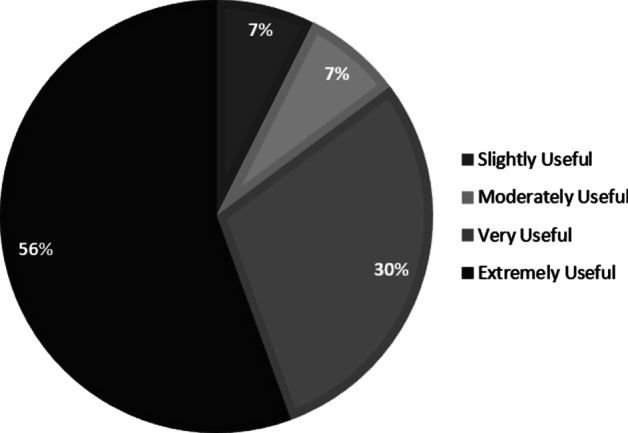
Percentage of students providing different ratings of usefulness for captions and transcripts. No students felt that these were not at all useful

Participants rated the usefulness of captions and transcripts when they had received them (1 = not at all useful, 5 = extremely useful). Figure 2 indicates that the majority viewed them as extremely or very useful, with no students indicating that they were not at all useful. When asked whether the availability of these would encourage them to make more effective use of lecture capture, there was no difference in responses between the CT + group (*M* = 4.44, *SD* = 0.87) and CT − group (*M* = 4.61, *SD* = 0.54), *t* (40) = − 1.417, *p* = 0.164, 95% CI [− 0.41, 0.07], *d* = 0.221.

For the effects of reminders, survey data revealed that there was no difference in terms of the number of topics for which capture was accessed (R + *M* = 2.23, *SD* = 1.64, R − *M* = 2.31, *SD* = 1.69), *t* (41) = 0.277, *p* = 0.783, 95% CI [− 0.59, 0.45], *d* = 0.043. Reminders also had no impact on the ease of using lecture capture (R + *M* = 4.68, *SD* = 0.48, R − *M* = 4.67, *SD* = 0.63, *t* (30) = 0.626, *p* = 0.536, 95% CI [0.10, − 0.28], *d* = 0.112), how much students perceived capture to support learning (R + *M* = 4.81, *SD* = 0.40, R − *M* = 4.81, *SD* = 0.40, *t* (30) = 0.000, p = 1.00, 95% CI [− 0.21, 0.21], *d* = 0.001) or whether they would recommend it to a friend (R + *M* = 4.87, *SD* = 0.34, R − *M* = 4.81, *SD* = 0.48, *t* (30) = 0.701, *p* = 0.489, 95% CI [− 0.12, 0.57], *d* = 0.126).

Table 4 shows the functions used by students according to their reminder grouping. As for CT grouping, most students were using the time bar to visit specific sections, pause and restart and adjusting speed but few utilised book marking or study notes. McNemar Chi-Square tests showed no differences in functionality between the two groups.

**Table 4:** Percentage of students using specific functions of lecture capture

Function (N)	R − (% using)	R + (% using)	Test statistic, *χ*^*2*^* (df)*	Significance
Time bar (38)	65.8	63.2	0.736 (1)	*1.00*
Pause/Restart (36)	78.1	78.4	4.592 (1)	*1.00*
Speed (37)	54.1	70.3	8.111 (1)	*0.109*
Bookmarking (37)	5.4	10.8	3.368 (1)	*0.625*
Study notes (37)	40.5	40.5	16.295 (1)	*1.00*

**Fig. 3:**
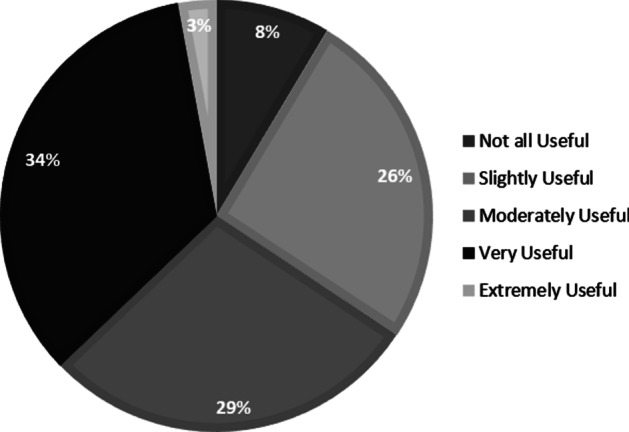
Percentage of students providing different ratings of usefulness for reminders

Students rated the usefulness of reminders when they had received them (1 = not at all useful, 5 = extremely useful). Figure 3 indicates that most students found the reminders very or moderately useful but around 10% found them to be not all useful. Given the reminders referred to functions of Echo360, the lecture capture software used, as well as specific content of the capture, students were asked separately whether the regular email reminders would encourage them to make more effective use of lecture capture content and functions. There was no difference in responses between those sent reminders (*M* = 3.92, *SD* = 0.93) and those not (*M* = 3.95, *SD* = 1.139), *t* (40) = − 0.453, *p* = 0.653, 95% CI [− 0.40, 0.25], *d* = 0.073 in terms of making effective use of content. Similarly, when asked whether the reminders about the different functions of lecture capture would encourage them to make more effective use of these functions, there was no difference in responses between those sent reminders (*M* = 3.52, *SD* = 1.18) and those not (*M* = 3.47, *SD* = 1.28), *t* (39) = − 1.612, *p* = 0.115, 95% CI [− 0.56, 0.06], *d* = 0.255.

#### The impact of transcripts and reminders on student performance

Data from the 42 students who completed both surveys was used to investigate the impact on performance. Paired sample t-tests demonstrated that there was no difference in the performance on the MCQ test between CT + (*M* = 5.90, *SD* = 1.86) and CT − (*M* = 6.13, *SD* = 1.90), *t* (41) = 0.696, *p* = 0.490, 95% CI [− 0.93, 0.45], *d* = 0.107. There was also no difference in performance when students received reminders (*M* = 6.33, *SD* = 1.56) and when they did not (*M* = 5.71, *SD* = 2.12), *t* (41) = 1.874, *p* = 0.068, 95% CI [− 0.05, 1.29], *d* = 0.289.

### Phase 2: qualitative outcomes

Thematic analysis identified four themes underlying participants’ views of lecture capture. The first theme—perceptions of the lecture as a learning tool—provided important contextual information, which although not directly related to our research aims, provides an explanatory backdrop for student use of LC. The remaining themes (perceptions of lecture capture as a learning aid; perceived value of lecture capture captions and transcripts; perceived value of reminders) directly relate to the use of LC and the independent variables assessed in the quantitative work. Given the indirect link between the first theme and the specific research question, we provide only a brief overview of this theme.

#### Perceptions of the lecture as a learning tool

Participants regarded lectures as information-dense and a core learning activity: *“Lectures are really helpful because they probably provide you with the most information.”* (Participant 3 [P3]). Other types of teaching were typically thought to play a consolidatory (“*Seminars are more important [than lectures]. I think they’re more useful because they involve a group session so you can get different viewpoints.*” P5) or supplementary role, although not always successfully (“*[the seminar] doesn’t supplement the material that we learned as much as we could have*” P1).

Participants felt it was crucial to have the lecture slides on the VLE in advance (“*It’s really important for me that before the lecture the slides are updated on [institutional VLE] so that I can I usually have a look through the slides before the lecture.*” P8) and that lecturers did not stray too much from the slides in their delivery. The volume of information on each slide appeared unimportant: “*I don’t mind like 50 slides or 100 slides, just have them […] simple and easy to understand and talk about what’s in it so it’s easier to keep track*” (P4). The use of text combined with graphic information was considered helpful: “*I like when there is some text on the slide [and] that there are not only pictures*” (P8).

Participants wanted lectures to be engaging with diverse content with videos and quizzes both identified as helpful (“*it’s really enjoyable when they use videos during the lecture personally, then, I think I remember the material from the lecture*” P8; “*At points throughout the lecture, we get like the questions […] which is good because it breaks the lecture up a little bit. And it also makes sure that you actually.*” P3). They noted that even when content is engaging, it should not be unnecessarily repeated (“*[Anecdotes] make the lecture more interesting, but sometimes they’re just like too many of them and then they're like, kind of repetitive*” P8) and that it must be delivered at an appropriate pace to allow for note-taking (*“I do need the time to take some detailed notes because I’m not very good at remembering the information content*” P7). Participants noted that aids to support note-taking were useful and could free them up to “*actually fully understand the topics*” (P3).

#### Perceptions of lecture capture as a learning aid

Typically, the perceived purpose of lecture capture was three-fold. Firstly, participants noted that it can help consolidate learning because it enables repeated viewing and pausing (“*Just listening to it more times makes it stick to memory*” P5). Secondly, lecture capture was perceived to aid notetaking and understanding where focus was lost during the lecture (“*it’s easy to switch off when you’re in the actual thing*” P6), or where the lecturer had spoken too quickly. Finally, it was used to compensate for non-attendance (“*I was absent for one of the lectures last term … I watched the whole lecture obviously to catch up*” P7). In this context, students found it useful for class discussions and quizzes to be captured in the recordings (“*it can be a disadvantage if you’re not there, because sometimes they turn it off [….] so you miss out on some of that information*” P6). When capture was used also varied. Some used it to inform notetaking or clarify concepts soon after the lecture, whilst others relied on capture primarily for revision before exams (“*I wouldn't watch the whole lecture ever again. But I do watch certain parts if I don’t understand something*” P2).

#### Perceived value of lecture capture captions and transcripts

Participants typically found captions and transcripts helpful. One reason for this was that they afforded efficient learning. Specifically, captions and transcripts, unlike audio content, were searchable, making it “*really easy to find certain points, and certain phrases*” (P1). Additionally, they offered a quicker means of reviewing content (“*you don’t have to keep rewinding the lecture capture, […] you can just read it. So that’s often a time saver and really useful*” P6).

A second reason participants felt that transcripts and captions were useful was that they offered another means of engaging with the lecture content especially when content was challenging or fast-paced (“*if I could see the transcripts up there I could [grasp it better]”* P4). Further, transcripts were considered an aid for foreign students and for those who share workspaces so may not be able to listen to audio files. Nonetheless, given that transcripts may contain incorrectly transcribed words, or may lack important context, participants felt they should be used alongside the audio (“*if I was just relying on the transcript, then it wouldn’t really be useful. So it means I have to listen.*” P5).

#### Perceived value of reminders

Despite reminders including details of key lecture content and capture functionality, participants typically focused only on the content component and did not comment on functions they utilised or noted from the email reminders (“*the first thing that I watched was actually those time intervals mentioned in the email. […] and it’s made like it encouraged me to spend more time on lecture capture*” P8). Participants suggested that receiving email reminders is not ideal because they receive a lot of emails and tend to ignore them (“*I remember getting reminders … [but] I get quite a lot of emails and a lot of the time I just don’t really look through them that much*” P2). Some participants suggested that reminders could be incorporated into other existing communications (e.g., forums) (“*we can […] incorporate into an existing process, not two separate ones*” P1). Participants also suggested weekly reminders may be too frequent (“*maybe once every two weeks [would be preferable].*” P2). They suggested that having reminders at the earlier stages of the module would be useful but beyond this, they saw little or no benefit of additional reminders. Furthermore, those who only used lecture capture occasionally, in a targeted fashion, did not find reminders useful: “*I didn’t access lecture capture unless I had a specific reason to like I’ve missed a lecture or during revision […] so I didn’t really use them*” P7).

## Discussion

When evaluating any tool designed to support lecture learning, it is important to recognise the context of the lectures and what they mean to students as well as the impact of the tool itself. The mixed-methods approach taken here allowed us to achieve this. The qualitative data aligned with results of previous studies; students indicated that they value lectures highly and perceive them as providing core knowledge and are impacted by the pace and clarity of presentations (Dommett et al., 2020). They recognise the need for note-taking during lectures and appreciate opportunities to engage in more active processes such as quizzes. Correspondingly, students perceived lecture capture to serve three main purposes that have been reported in previous research: consolidation through self-paced review of the material (Al-Nashash & Gunn, 2013); as a note-taking aid (Dommett et al., 2020; Gosper et al., 2010; Newton et al., 2014; Saunders & Hutt, 2015); and to substitute for non-attendance (Edwards & Clinton, 2018). Given these similarities with other research, we can be confident that the cohort sampled here hold typical views of lectures and capture.

Despite the high value placed on lectures and captures, system analytics data across the entire participant group showed a relatively low number of lecture capture video views and percentage of video viewed. Furthermore, this decreased between the first and second half of semester. This suggests that despite students valuing lecture capture highly, they did not appear to make extensive use of it. There are likely to be several reasons for this. Firstly, previous research has consistently shown capture use is highest during revision periods (Brady et al., 2013; Gosper et al., 2010) but our data was collected before the revision period. Secondly, this module runs in the first semester of the first year at university and attendance is typically quite high at live lectures which may reduce the need to use LC to substitute for attendance. Thirdly, although many students on the module will not have previously studied Social Psychology, students typically gain high grades on this module (previous year data average grade 64%; upper second classification), in contrast to the other core modules which run alongside it. This could suggest that there they are more comfortable with this content and do not need to revisit it as often to consolidate learning or take notes. Module feedback also suggests that the pace of delivery for this module is appropriate, which may again reduce the need for capture views. As such analytics data on other modules may find higher usage of LC in contrast to that reported here.

### The impact of captions, transcripts and reminders

In terms of the effects of captions and transcripts on analytics, there was no effect in the first half of the semester, Time 1 (Topics 1–5), but students with access to these in the second half of the semester, Time 2 (Topics 6–10) showed greater capture engagement in terms of both the number of views and the percentage of the video viewed. Given that we studied first-year undergraduates in their first semester, it is possible this difference emerges as students become more comfortable with the technology and settle into university study. Survey data from a subset of students showed that caption and transcript availability across the module did not affect the number of topics for which they accessed the capture and the ease with which they used it. There was a trend towards significance for the perceived use of lecture capture to support learning with those receiving captions and transcripts giving a higher perceived usefulness score. Qualitative data indicated that students found the captions and transcripts helpful in consolidating their learning. Students cited the use of combined visual and audio cues, in line with the Cognitive Theory of Multimedia Learning which suggests the dual processing of visual and verbal information enhances learning. The written text was used to help identify specific sections because it was searchable. The associated transcript was also considered helpful in supporting use of specific terminology. As has been found previously, the use of captions and transcripts was thought to be particularly helpful when students received teaching in a second language (Kent et al., 2018) or the lecturer had a heavy accent (Tisdell & Loch, 2017). Given the positive reports of the use of captions and transcripts from the qualitative work, it is perhaps unsurprising that students with access to these were more likely to recommend capture to a friend. Use of the different functions within lecture capture was similar across both groups with students most commonly using the time bar to navigate to specific places, pausing and restarting and adjusting the speed. The common use of these functions is in line with previous research showing similar tool usage (Dommett et al., 2020). Despite caption and transcript availability not resulting in more lecture captures being accessed, most considered these useful, although they did not believe they helped them make more effective use of capture. MCQ tests revealed no difference in performance with the availability of captions and transcripts. This is at odds with previous studies which have indicated both benefits (Taylor, 2009) and adverse effects (Ritzhaupt et al., 2015).

Analytics showed no effects of reminders on the number of videos viewed or the percentage viewed. This was reflected in the survey data which revealed that students accessed the same number of topics irrespective of whether they received reminders. The reminders, which included links to help pages on using lecture capture had no impact on ease of using the capture or how much they perceived capture as supporting learning. There was also no impact on whether they would recommend capture to a friend.

Given the reminders included reference to the specific functions, we might have expected to see an increase in function use with reminders. This was not the case. Unlike captioning and transcripts, student perceptions of the utility of the reminders were lower and the data suggests no impact of the reminders on the ability to use content or functions more effectively. There was also no impact on MCQ performance. Previous research has shown that reminder emails can increase weekend study time, cognitive effort during study and exam performance (O’Connell & Lang, 2018). We see no improvement in the MCQ tests here, but it is possible later effects would have emerged in a real examination. The qualitative data provides some insight into the lack of effects. For example, students reported getting too many emails to read them properly or using the content of the email as an indicator of what topics to review rather than how to review them. It was also noted that unless they particularly needed to review a lecture, these reminders were ignored.

### Limitations of the current study

The current study is not without its limitations. The study is a case study and therefore, by definition, generalisability to other institutions or contexts may be limited. However, given the similarities to previous research in the view of lectures and lecture capture, it is logical to assume that the students sampled here use lectures and their capture in a manner reflective of the wider student body. Secondly, we were unable to separate out captions and transcripts in our lecture capture system meaning we cannot be certain exactly which feature students considered during the survey, although qualitative data suggests both were considered and used. Thirdly, our design meant that it was not possible to match quantitative and qualitative data and therefore identify key characteristics (e.g., amount of LC use) of those interviewed. However, the data from both methods align well and this approach reduced the likelihood of students being unwilling to be interviewed because their performance or ability would be known by researchers. Fourthly, the sample size of both the survey and interviews is relatively low. The crossover design means a smaller sample size is less of a problem than a between measures design and the lower number of interviews arose due to reasons beyond our control (COVID-19). Fifthly, the performance measure used in the present study were collected specifically for the study. Students were advised that completing them was good practice for the real MCQ examination and the questions were designed to measure improvement against the learning outcomes in the exact same way as exam questions but, of course, students were likely to view them quite differently. They may not have considered these important or beneficial to engage with and this could have impacted on their performance. Given the ethical constraints imposed by the host university, which dictated that all groups must have access at some point to something that could potentially enhance module performance, this limitation was unavoidable. Finally, this research was conducted prior to the COVID-19 pandemic. The subsequent pandemic may have impacted on the way that students study online which could mean they have changed their views or approaches since those reported here. However, it is likely that lectures and lecture capture will still have some place in post-pandemic teaching, probably as a blended approach (Maguire et al., 2020) meaning these findings are still of value. Furthermore, students may have become more digitally-able through greater digital immersion (Helsper & Eynon, 2010) during the pandemic, increasing the likelihood that they will engage with more functionality of lecture capture in future and therefore, making it even more critical that the impact of additional functions is understood.

### Future research

Future studies should attempt to address the limitations outlined above and extend the study to a broad range of disciplines and levels of study. Research suggests that lectures and capture is used across disciplines (Dommett et al., 2020) but there may be differences in terms of the utility of captions, transcripts and reminders. Furthermore, future research should be contextualised in the post-pandemic lecture. Exactly what this will look like is not yet fully known but, blended learning is predicted to be the most pragmatic and desirable approach in providing education in the future (Dua et al., 2020). This is the approach being adopted for the module examined in the current case study. Therefore it may be of value to consider the worth of caption and transcripts on pre-recorded lecture videos, which are currently available via a different software. A blended learning approach is also likely to support widening participation efforts within HE (Jones & Lau, 2010), which means that any tools that can support inclusivity and accessibility should be carefully reviewed. One of the most popular blended approaches is the flipped classroom in which students view lecture videos or other resources in advance and come onto campus for more interactive sessions (Bergmann & Sams, 2012). This approach has been used throughout the pandemic (Martin, 2020; Perez & Mirabent, 2020) but no analysis has been conducted on use captions, transcripts and reminders specifically.

### Conclusions

Despite the limitations of the current research, the present study confirms previous findings regarding the value students place on lectures and their capture. Further it demonstrates that students value the availability of captions and transcripts, and their usage appears to align with Cognitive Load Theory of Multimedia Learning. Although valuing use of these, there were no overall differences in performance or beliefs about lecture capture when these were available. In contrast to the captions and transcripts, push reminders were not valued by students and had no significant impact on any measures, suggesting there may be less value in pursuing these. Certainly, as universities face difficult decisions in the light of the COVID-19 pandemic on where to focus resource, reminders may be of less value than ensuring captions and transcripts are available and accurate. In conclusion, the current study indicates that the use of captions and transcripts is perceived as useful by students and explanations given suggest the multimedia components combining visual and auditory input suggesting that this functionality should be included in videoed lectures in future.

## Supplementary Information

**Additional file 1.** Supplementary Information 1 – Module Learning Outcomes.

**Additional file 2.** Supplementary Information 2 – Interview Schedule.

## Data Availability

The datasets used and/or analysed during the current study are available from the corresponding author on reasonable request.
