# Supplementary material for: Effects of captions, transcripts and reminders on learning and perceptions of lecture capture
Source: Int J Educ Technol High Educ. 2022 Apr 26;19(1):20. doi: 10.1186/s41239-022-00327-9 (PMC9038223; doi:10.1186/s41239-022-00327-9)
Supplement: Supplementary file 1 — Additional file 1. Supplementary Information 1 – Module Learning Outcomes. [file 41239_2022_327_MOESM1_ESM.docx]

# Supplementary Information 1 – Module Learning Outcomes

The module used for this research had the following over-arching module learning outcomes, that by the end of the module, you should be able to:

1. Appreciate the history and development of social psychology

2. Describe, compare, and contrast key social psychological theories and approaches, including evolutionary psychology

3. Understand the relationship between social psychology and other areas of psychology

4. Understand and discuss social psychological research methods, including qualitative and quantitative methods; and appreciate the strengths and limitations of different methods

5. Understand, and describe in language accessible to the lay public, key topics in social psychology including attitudes, attribution, intra- and inter-group behaviour, conformity, persuasion and social identity

6. Apply theories and concepts from social psychology to describe and explain key contemporary issues such as racism and conflict

In addition to these over-arching outcomes are specific topic learning outcomes associated with each of the ten topics covered. For example, for Topic 1 (The Self), after completing this topic and associated activities, students should be able to explain:

1.1 Describe theories and research around self-schemas and the self-concept, the functions they perform, and how they may affect behaviour

1.2 Explain how ‘possible selves’ act as a source of everyday motivation

1.3 Explain how private and public self-awareness may be activated, and the behavioural implications of activating self-awareness

1.4 Describe psychological processes through which we may acquire information about ourselves from: (a) others (b) the media (c) observing ourselves

1.5 Define, and explain why people engage in, 'impression management'

An example of an MCQ question assessing learning of this topic is:

In Beaman et al. (1979) study, children who had unrestricted access to a bowl of sweets were more likely to follow instructions and take only one sweet where the bowl was placed next to a mirror. Which of the following forms of self-awareness were likely activated by the presence of the mirror?

- Private self-awareness only
- Public self-awareness only
- Both private and public self awareness
- Neither private nor public self awareness
