# Supplementary material for: Effects of captions, transcripts and reminders on learning and perceptions of lecture capture
Source: Int J Educ Technol High Educ. 2022 Apr 26;19(1):20. doi: 10.1186/s41239-022-00327-9 (PMC9038223; doi:10.1186/s41239-022-00327-9)
Supplement: Supplementary file 2 — Additional file 2. Supplementary Information 2 – Interview Schedule. [file 41239_2022_327_MOESM2_ESM.docx]

| Topic | Questions |
| --- | --- |
| Lectures | Which format do you find is the most useful for you to learn? Follow-up: Why? |
|  | What makes a good lecture for you?  How do you work through lectures, e.g. note-taking? |
| Lecture capture | Did you use lecture capture in the X module? Follow-up: What prompted you to use it? |
| Transcripts | Some Lecture Capture recordings have written transcripts available. What do you think of these? |
|  | What impact, if any, do you think lecture capture transcripts have had on studying and learning? |
|  | Based on your experience, do you think anything can be improved about the lecture capture transcripts? |
|  | If you had to persuade somebody in senior management that lecture capture transcripts are worthwhile, what would you say to them? |
|  | When you have the transcripts, what process would you go through in terms of using the transcripts? |
|  | Would you rely on these transcripts if they were accessible to you? |
| Reminders | You were receiving email reminders for a period about access to Lecture Capture and which identified a couple of key areas that may be appropriate to review. How did you feel about these? |
|  | Have you spoken to any of your peers about the use of reminders or the short key information? |
| End | Is there anything else that you would like to add on the topic of transcripts? |

# Supplementary Information 2 – Interview Schedule
